# Critical reflections on the impact of late‐life social activity on dementia and mild cognitive impairment (MCI)

**DOI:** 10.1002/alz.70108

**Published:** 2025-03-27

**Authors:** Sijia Liu, Jialao Ma

**Affiliations:** ^1^ School of Law and Intellectual Property Guangdong Polytechnic Normal University Guangzhou China; ^2^ School of Law Sun Yat‐sen University Guangzhou China; ^3^ The Affiliated Guangzhou Twelfth People's Hospital Guangzhou Medical University Guangzhou China; ^4^ School of Public Health Guangzhou Medical University Guangzhou China

**Keywords:** biological pathways, dementia, longitudinal data, mild cognitive impairment (MCI), multiple imputation, qualitative methods, social activity, time‐dependent Cox model, virtual social interaction

1

Dear Editor,

We were particularly intrigued by the recent article by Chen et al., which is titled “Late‐life social activity and subsequent risk of dementia and mild cognitive impairment.”^1^ This paper provides insightful opinions on the relationship between late‐life social activity and dementia risks/mild cognitive impairment (MCI). Nonetheless, we would like to mention several limitations:

First, the study concentrates predominantly on measuring the quantity and frequency of social activities, without enough concerns for evaluating the quality or emotional importance of these interactions.^2^ For instance, meeting with a social group may have far less significant perceived value than conversations with relatives or close friends. Such glaring differences between superficial and deep social experiences may constrain the explainability of results. Thus, we suggest using qualitative methods like structured interviews or validated scales to assess the emotional and cognitive impact of social activities.^3^ This will enable a clearer understanding of how different types and qualities of social interactions affect cognitive health.

Second, the study provides only a brief description of the potential mechanisms whereby social activity may benefit cognitive health, like cognitive stimulation, stress reduction, and neurogenesis, without directly exploring these pathways in their research.^4^ This could raise concerns on the interpretability concerning the mechanisms through which social activities could help lessen or even prevent the onset of dementia. Hence, future studies may extend such links through the incorporation of biomarkers like neuroimaging, cortisol levels, or inflammatory markers to investigate the potential biological pathways inside. Moreover, despite the study controlled for loneliness and depression, these variables could themselves be bi‐directionally related to social activities and dementia (e.g., depression may yield decrease in social activities and regarded as an early alarm of dementia).^5^ Such variables could be investigated as mediators/moderators so as to enhance the better understanding of the relationship between social engagement and the risk of dementia.

Third, this study appears to lack longitudinal data, which could potentially reflect the changes in social activities over time.^6^ Because social activity patterns are very likely to be altered due to factors such as health, aging, and living environment, solely measuring social activity at baseline may not precisely capture the broader and dynamic relationship that it may have with dementia risk. For example, elderly people who were very socially active may, on account of some chronic diseases, become less involved, thereby increasing their chances of getting dementia. Furthermore, the shift toward greater use of virtual social interaction, particularly since the coronavirus disease 2019 (COVID‐19) pandemic, compels us to examine the impact of digital tools on cognitive health.^7^ We think that conducting retrospective studies on pandemic‐era data or incorporating digital engagement measures into future studies could benefit more for dynamic social activity measurement. In addition, using objective measures, such as wearable devices that detect social interactions (e.g., time spent in proximity to others), could complement self‐reported data effectively.

Notably, the authors rely largely on the Cox proportional hazards model and the accelerated failure time (AFT) model, both of which are based on the linear relationships between variables and time independence, whereas these models seem to disregard the dynamic characters of certain variables like social activity. To counter this challenge, we recommend adopting a time‐dependent Cox model, which defines social activity as a time‐dependent variable, making it possible to influence throughout the follow‐up period.^8^ In addition, the progression of dementia is usually considered in stages (e.g., no cognitive impairment–MCI–dementia).^9^ It is evident that a single‐event Cox model will not fully present these changes in transition. Therefore, we propose the use of multi‐state models to explore the dynamic transitions across these stages. Such an approach would provide a more thorough way of assessing the impact of social activity.

Finally, the authors mentioned that 10% of participants (197 people) had missing baseline data for variables like social network size, social support, or loneliness. To address this, they performed a complete case analysis, which excluded participants with missing data. However, this may increase the bias risk and reduce the entire statistical power if the missing data are not random. To solve this, we suggest using multiple imputations to fix this tough issue.^10^ By simulating missing values multiple times to create complete datasets, this approach may enhance the robustness as well as the accuracy of results while maximizing data utilization.

In conclusion, the study of Chen et al. symbolizes important contributions to the understanding of how late‐life social activity can mitigate the risk of dementia and MCI. By addressing these aforementioned aspects, future research could add more value to the clearer understanding of social activities in preventing, reducing, or delaying dementia.

## AUTHOR CONTRIBUTION

*Conceptualization*: Sijia Liu. *Refinement* – *original draft*: Sijia Liu. *Revising*: Sijia Liu. *Suggestion*: Jialao Ma. *Writing*: Sijia Liu and Jialao Ma.

## CONFLICT OF INTEREST STATEMENT

We declare no competing interests. Author disclosures are available in the Supporting Information.

## ETHICS STATEMENT

Not required – being a theoretical paper.

## Supporting information



Supporting Information
